# Impact of Diet Composition on Insulin Resistance

**DOI:** 10.3390/nu14183716

**Published:** 2022-09-09

**Authors:** Fátima O. Martins, Silvia V. Conde

**Affiliations:** iNOVA4Health, NOVA Medical School, Faculdade de Ciências Médicas, Universidade Nova de Lisboa, Campo Mártires da Pátria, 1169-056 Lisboa, Portugal

Insulin resistance is a complex condition in which the body does not respond adequately to insulin, a hormone secreted by the pancreas with an essential role in the regulation of blood sugar levels. This condition is one of the major factors in the pathology of cardiometabolic diseases, which are commonly associated with peripheral insulin resistance. Peripheral insulin resistance consists of an impaired biologic response to insulin stimulation of peripheral target tissues, namely the liver, muscle and fat tissue [1]. More recently, central insulin resistance has been highlighted as fundamental also in cardiometabolic diseases since insulin plays an important role at brain circuitries that control food behavior and autonomic activity [1,2]. Moreover, brain insulin resistance is associated with cognition impairment and important neurodegenerative diseases such as Alzheimer’s Disease and Parkinson Disease [3].

Several genetic and lifestyle factors can contribute to insulin resistance, with disruptions in diet composition being one of the major factors contributing to this condition. In contrast, different feed regimens and some nutrients have beneficial impacts on insulin resistance and disease development.

This Special Issue was developed to compile studies that highlight the beneficial or deleterious impact of different nutritional plans on insulin sensitivity and metabolism and that unravel mechanistic links between diet composition and nutritional status and the development of insulin resistance, both peripherally and centrally.

One of the most consumed food components worldwide is caffeine. This xanthine is the most widely consumed psychoactive substance in the world and is present in several dietary sources regularly consumed, such as tea, coffee, cocoa beverages, chocolate bars, and soft drinks [4]. In recent decades, physicians have advised hypertensive and diabetic patients to limit caffeine intake, based on several studies stating that caffeine acutely increases blood pressure [5] and lowers insulin sensitivity [6,7,8]. More recently, chronic coffee/caffeine intake was associated with an improved insulin sensitivity and glucose metabolism [9,10] and a lower risk of type 2 diabetes [11], clearly suggesting that acute and chronic caffeine intake have opposite effects on metabolism. However, obstructive sleep apnea (OSA) patients that frequently exhibit cardiometabolic dysfunction and insulin resistance [12,13] consume on average three times more caffeine than control subjects [14]. In this issue, Conde and colleagues [15] studied the impact of caffeine intake on OSA severity and OSA association with dysmetabolism and sympathetic nervous system dysfunction. They found that OSA patients consume more caffeine, but this was not associated with OSA severity or with dysmetabolism and sleep fragmentation, which rejects the common clinical recommendation for caffeine avoidance in OSA patients. The only parameter that these authors highlighted to be somehow disrupted by high caffeine levels in OSA patients, and therefore should be taken in account for clinical recommendations, is the overactivation of the sympathetic nervous system, which is frequently associated with some cardiovascular conditions [16,17] but also with insulin resistance.

Several different diet patterns are being adopted by an increasing proportion of the world population. Diet patterns can be defined as the quantities, proportions, variety, or combination of different foods, drinks, and nutrients in diets, and the frequency with which they are habitually consumed [18]. While some of these have been described to have deleterious impacts on health, e.g., hypercaloric diets [19], others have been described as beneficial, e.g., the Mediterranean diet [20]. However, the effects of such diet patterns on physiology and pathology are surrounded by controversy due to the different results between populations and pathological conditions. For example, discussions about the impact of different regimens for the prevention of chronic diseases related with insulin resistance such as type 2 diabetes, obesity, and non-alcoholic fatty liver disease (NAFLD) among others, form the basis of a number of studies that can be found in the literature.

In this Special Issue, this discussion occupies an important space. Banaszak and colleagues reviewed the positive effects of vegetarian and vegan diets on insulin resistance [21]. These authors concluded that vegetarian and vegan populations have better blood parameters, and that more plant-based foods and fewer animal foods in a diet result in lower insulin resistance and a lower risk of prediabetes and type 2 diabetes. Additionally, they discussed the possible use of these plant-based diets in clinical applications for the treatment and prevention of chronic diseases since parameters such as body weight, body fat, BMI and lipid profile improve under this type of diet. They also showed that meat-free diets are suitable for everyone, regardless of age or health, but improperly balanced plant-based diets may carry a risk of nutritional deficiencies; in particular, deficiencies in protein, B vitamins, iron, zinc, and omega 3 fatty acids have been noted. They recommend further clinical research and provide guidance on future research directions.

Another study in this Special Issue discussing the impact of a plant-based diet on insulin resistance was conducted by Lorinczova and colleagues [22]. In this original study, the authors tested the impact of plant-derived proteins from rice, potato and of whey on insulin secretion, glucose maintenance and appetite perception in a group of healthy males in a single-blind, randomized study. They found differing glycemic and insulinemic properties between potato, rice and whey proteins following ingestion, with whey promoting a higher increase in insulin and GLP-1 secretion with a consequent higher decrease in glucose levels than plant-derived proteins. Moreover, dampened insulin and GLP-1 responses with better glycemic regulation after the ingestion of plant proteins compared to whey, with no significant differences in average appetite perception, was observed. Taken together, the results of the study suggest that the characteristics of each protein, irrespective of plant or animal origin, result in differing metabolic responses and that plant-based protein regimens may have benefits for populations where tighter control of glycemic and insulinemic regulation may be beneficial while maintaining total protein intake.

It is agreed upon that high sugar intake is associated with insulin resistance and with an increased incidence of metabolic diseases, such as obesity and metabolic syndrome. Sugars can be categorized as intrinsic/natural and extrinsic/added sugars depending on if they are naturally present in the structure or matrix of whole fresh fruits and vegetables, milk, and dairy products without further processing, or if they are added to food. Added sugars include sucrose, fructose, glucose, starch hydrolysates and other isolated sugar preparations added during food preparation and manufacturing [23]. These intrinsic and added sugars have been described to have different impacts on these pathological conditions, with the added sugars being highly associated with metabolic diseases. However, there is still controversy about whether the intrinsic sugars from fruit juices have a similar harmful effect as sugars added to beverages. The work of Monteiro-Alfredo and colleagues [24] contributes to the knowledge in this field by comparing the impact of four different fruit juices administered across four weeks with sugary solutions having a similar sugar profile and concentration on weight, hyperglycemia, glycation and oxidative stress in control and diabetic animal models. They demonstrated that sugars naturally present in fruit juices have a less severe impact in terms of metabolic control than the added sugars in foods that promote a poorer glycemic profile and increased levels of glycation and oxidative stress, particularly in tissues such as the heart and the kidney [24]. Taken together, these results reinforce the evidence supporting a noxious role for added sugars and a harmless effect of moderate intakes of fruit sugars, even in diabetic models. Nevertheless, the authors stress that more research should be performed to investigate the long-term effect of fruit juices in metabolism as well as in animal models.

A comparison between two carbohydrates, isomaltulose—a disaccharide carbohydrate composed of glucose and fructose—and sucrose, in arterial stiffness in response to acute hyperglycemia was also performed in this Special Issue by Kobayashi et al. [25]. With the knowledge that an increased arterial stiffness in response to acute hyperglycemia is associated with high cardiovascular risk [26], this study investigated the efficacy of low-glycemic-index isomaltulose on arterial stiffness during hyperglycemia in ten middle-aged and older adults. They found that arterial stiffness and systolic blood pressure did not change following isomaltulose intake in middle-age and older adults, in contrast with sucrose ingestion, suggesting that isomaltulose could be used as an alternative to sucrose.

The Mediterranean diet has been referenced for decades as a diversified diet with a beneficial impact on cardiometabolic diseases. Interest in this diet began in the 1950s/1960s, when it was realized that Mediterranean countries had lower rates of heart disease than other countries worldwide [20]. However, the impact of the Mediterranean diet is broader as there is robust evidence suggesting that it improves HbA1c and insulin sensitivity [27,28], which are benefits that have been associated with the presence of large number of functional foods and nutraceuticals. Such a diet, predominantly plant-based, is characterized by a high consumption of extra-virgin olive oil, nuts, red wine, vegetables, and other polyphenol-rich elements, with a moderate consumption of fish, poultry, and eggs and a low consumption of red meat [20]. High-protein diets have also been shown to be efficacious in promoting weight loss along with improvements in insulin sensitivity [19]. The comparison between a diversified diet pattern, such as the Mediterranean diet, and a high protein diet can be observed in the original article from Tettamanzi et al. [29] where they found that a high protein diet was more effective in reducing insulin resistance and improving glycemic control in morbidly obese women with pre-diabetes. Additionally, they assessed microbial diversity in the gut of these women, and identified a panel of microbes that explain the differences in the effect of the two diet patterns. However, further investigation is required to elucidate the links between dietary interventions, the microbiome and insulin action regulation.

Still related to products from Mediterranean countries, Azab and colleagues reviewed the impact of carob, one of the major food trees for people of Mediterranean origin, on the regulation of metabolism [30]. They described the nutritional composition of carob, highlighting that D-Pinitol as one of the most important components. D-pinitol has been used for decades as a medicinal product with antidiabetic, anti-Alzheimer, anticancer, antioxidant, anti-inflammatory, and immune- and hepato-protective properties. The authors state that more studies are needed to define the exact mechanisms of D-pinitol in insulin regulation as well as to establish the clinical applications of this compound and others found in carob.

Along with the beneficial impact of some diet components and patterns on metabolism [e.g., potato and rice—[22]] and the deleterious effects of others [e.g., added sugars—[24]] described in this Special Issue, the original work of Mohamad Hizami et al. introduces new results on the impact of probiotics on hepatic steatosis, fibrosis and biochemical blood tests related with NAFLD [31]. Probiotics are foods or supplements containing live microorganisms aiming to maintain or improve microbiota. Given that insulin resistance relates to the increased incidence of NAFLD and that the gut microbiota, by being part of the gut-liver axis, can be a target for NAFLD related problems, the authors performed a randomized, double-blind, placebo-controlled trial with 39 NAFLD patients supplemented with either a probiotics sachet (MCP^®^ BCMC^®^ strains) or a placebo for a total of 6 months. They did not find clinical improvements in NAFLD patients after the use of probiotics, but this treatment was shown to stabilize the mucosal immune function. These results led them to state that probiotics usage can protect NAFLD patients against the increase in intestinal permeability and suggest the need of additional studies with larger sample sizes, a longer duration, and different probiotic strains to evaluate the real benefit of probiotics in the management of NAFLD.

The impact of diet patterns on the regulation of the metabolism and in the pathological conditions related with insulin resistance is also known to be dependent not only on different cultural behaviors between different populations but also on their genetic background. Pandya and colleagues studied the Asian Indian (AI) population from Mangal Mandir, a Hindu temple in the Baltimore/Washington Metropolitan Area [32]. AI populations are at an increased risk of developing type 2 diabetes mellitus compared to other ethnic groups, even though they have a lower body mass index, for a number of reasons. These include that the age of onset for T2DM in AI populations is estimated to occur 10 years earlier than in Europeans; AI populations require lower BMI cut-offs for the effective identification of T2DM risk; and AI individuals may be predisposed to IR and T2DM because AI children are born smaller, have more fat, and less lean muscle [33]. The authors performed a descriptive statistical analysis where they found that weight loss may not be the recommendation for diabetes management in this population, yet an increase in protein and insoluble fiber consumption could play a critical role.

Tucker studied the relationship between several macronutrients and insulin resistance in 5665 non-diabetic U.S. adults and the author determined the extent to which these associations were influenced by multiple potential confounding variables [34]. The study was a cross-sectional design of 8 years of data from 2011 to 2018 from the U.S. National Health and Nutrition Examination Survey (NHANES) database. The author found that macronutrient intake was predictive of insulin resistance in this population with higher intakes of carbohydrates, leading to a worse scenario in terms of insulin resistance. Additionally, the author described that a higher intake of protein or unsaturated fat leads to lower levels of insulin resistance.

Additionally, in this Special Issue the thematic of methodologies to assess metabolic dysfunction is debated. The oral glucose tolerance test (OGTT) is recognized as the gold standard test for diagnosing diabetes [35]. However, even though it provides important information about glucose tolerance, it does not replicate the physiological effect of a complete meal and the impact of it this insulin secretion and insulin action [36]. Therefore, Lages and colleagues [37] reviewed clinical data that shows the importance of a mixed meal tolerance test (MMTT) as a method that is more reliable and better resembles physiological prandial changes when diagnosing metabolic alterations. They concluded that a complete nutritional challenge performed in the MMTT seems to be more physiological but the divergency in results highlights the need to compare this method and the OGTT in the diagnosis of diseases such as type 2 diabetes in larger clinical trials.

Finally, and with the knowledge that insulin does not only act in the periphery but is also present in the brain where it has an important role in the regulation of food behavior and cognition, this Special Issue also highlights the impact of central insulin resistance in the link between metabolic and neurodegenerative diseases.

Rafiee and colleagues debated the role of taurine in neurodegeneration-metabolic diseases [38]. Taurine is a sulfur-containing amino acid naturally found in meat, fish, dairy products, and human milk, and is also available as a dietary supplement. While it is generally accepted that taurine supplementation has beneficial effects in the peripheral dysmetabolism in animals and humans [39,40,41], several studies have also proposed that the deregulation of brain taurine homeostasis may play a role in dysmetabolism-neurodegeneration. Although taurine concentration is decreased in the brains of models of neurodegenerative disorders [42], diet-induced obesity leads to taurine accumulation in the hippocampus [43]. Therefore, the authors speculate that the cerebral accumulation of taurine might constitute a compensatory mechanism that attempts to prevent neurodegeneration, due to its cytoprotective effect. By reviewing the literature, they debated the role of cerebral taurine in obese and diabetic individuals and concluded that taurine contributes to brain health improvements in those subjects by various mechanisms including the modulation of inhibitory neurotransmission, the stimulation of antioxidant systems, and the stabilization of mitochondria and thus of energy production and Ca2+ homeostasis. They also suggest that further studies are needed to unravel the exact mechanisms of taurine action in metabolic disorders with an impact on brain function.

Finally, the study by Capucho and colleagues reviews the literature supporting the link between metabolic syndrome and neurodegeneration [44]. They discuss the impact of aging and dietary habits as important hallmarks for the development of neurodegeneration, focusing on two of the most prevalent neurodegenerative diseases, Alzheimer Disease and Parkinson’s Disease. They provide evidence for a central role of insulin in cognitive function regulation and food behavior control and review the mechanisms behind hypercaloric diet intake and brain insulin deregulation on neurodegenerative processes. They conclude by proposing new targets of therapeutic interventions to control these epidemics of metabolic and neurodegenerative diseases.

The studies compiled in this Special Issue are representative of the numerous studies focusing on the association between diet composition, dietary patterns and insulin resistance that have been conducted to date with controversial results. Therefore, more investigations are certainly needed to untangle the complex associations between diet composition and insulin resistance, both peripherally and centrally, to better understand the mechanisms behind the connection between different diet patterns and the development of metabolic and neurodegenerative disorders and to establish new methodologies to correctly diagnose these pathologies.

This Special Issue will open new doors to manage insulin-resistance associated diseases by appropriately and individually modulating nutritional strategies.
